# *cyclo*-Tetra­kis(μ-3-acetyl-4-methyl-1*H*-pyrazole-5-carboxyl­ato-κ^4^ *N* ^2^,*O* ^3^:*N* ^1^,*O* ^5^)tetra­kis[aqua­copper(II)] tetra­deca­hydrate

**DOI:** 10.1107/S1600536811030832

**Published:** 2011-08-17

**Authors:** Sergey Malinkin, Irina A. Golenya, Vadim A. Pavlenko, Matti Haukka, Turganbay S. Iskenderov

**Affiliations:** a Kiev National Taras Shevchenko University, Department of Chemistry, Volodymyrska Str. 64, 01601 Kiev, Ukraine; b University of Joensuu, Department of Chemistry, PO Box 111, FI-80101 Joensuu, Finland

## Abstract

The title compound, [Cu_4_(C_7_H_6_N_2_O_3_)_4_(H_2_O)_4_]·14H_2_O, a tetra­nuclear [2 × 2] grid-type complex with *S*4 symmetry, contains four Cu^II^ atoms which are bridged by four pyrazole­carboxyl­ate ligand anions and are additionally bonded to a water molecule. Each Cu^II^ atom is coordinated by two O atoms of the carboxyl­ate and acetyl groups, two pyrazole N atoms of doubly deprotonated 3-acetyl-4-methyl-1*H*-pyrazole-5-carb­oxy­lic acid and one O atom of a water mol­ecule. The geometry at each Cu^II^ atom is distorted square-pyramidal, with the two N and two O atoms in the equatorial plane and O atoms in the axial positions. O—H⋯O hydrogen-bonding interactions additionally stabilize the structure. One of the uncoordinated water molecules shows half-occupancy.

## Related literature

For the use of pyrazolate ligands in the preparation of polynuclear supra­molecular compounds, see: Piguet *et al.* (1997 ▶); Krämer *et al.* (2002 ▶); Zhang *et al.* (1996 ▶); Van der Vlugt *et al.* (2008 ▶); Klingele *et al.* (2007 ▶); Kovbasyuk *et al.* (2004 ▶); Pons *et al.* (2003 ▶). For the use of asymmetric ligands in the preparation of heterometallic complexes, see: Moroz *et al.* (2010 ▶). For related structures, see: Mokhir *et al.* (2002 ▶); Sliva *et al.* (1997 ▶); Wörl *et al.* (2005*a* ▶,*b* ▶); Świątek-Kozłowska *et al.* (2000 ▶). For the preparation of related ligands, see: Sachse *et al.* (2008 ▶). 
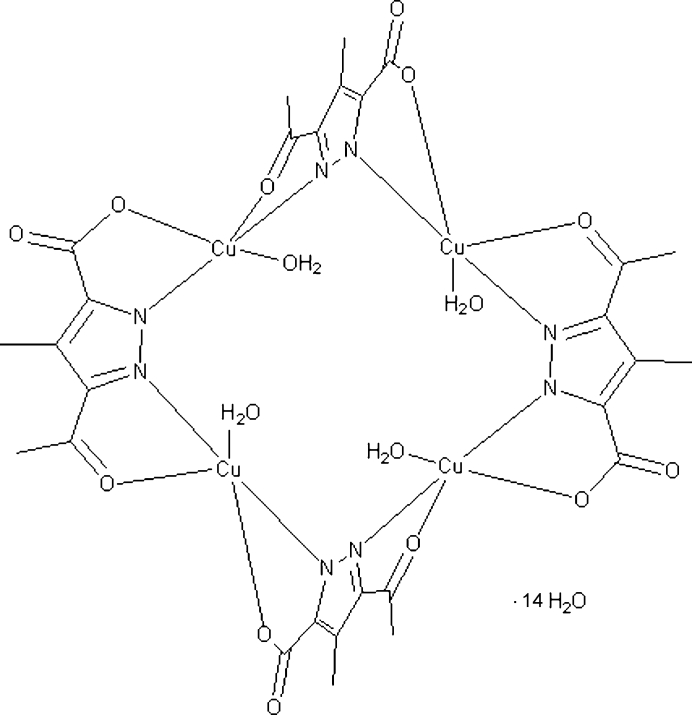


## Experimental

### 

#### Crystal data

[Cu_4_(C_7_H_6_N_2_O_3_)_4_(H_2_O)_4_]·14H_2_O
*M* *_r_* = 1243.00
Tetragonal, 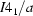

*a* = 13.8502 (7) Å
*c* = 26.280 (3) Å
*V* = 5041.1 (6) Å^3^
*Z* = 4
Mo *K*α radiation
μ = 1.76 mm^−1^
*T* = 100 K
0.35 × 0.25 × 0.15 mm

#### Data collection

Bruker SMART APEXII CCD diffractometer
Absorption correction: multi-scan (*SADABS*; Bruker, 2009 ▶) *T*_min_ = 0.578, *T*_max_ = 0.778
37816 measured reflections
3993 independent reflections
3254 reflections with *I* > 2σ(*I*)
*R*_int_ = 0.037

#### Refinement

*R*[*F* ^2^ > 2σ(*F* ^2^)] = 0.034
*wR*(*F* ^2^) = 0.097
*S* = 1.06
3993 reflections
165 parameters
H-atom parameters constrained
Δρ_max_ = 1.20 e Å^−3^
Δρ_min_ = −0.58 e Å^−3^

### 

Data collection: *APEX2* (Bruker, 2009 ▶); cell refinement: *SAINT* (Bruker, 2009 ▶); data reduction: *SAINT*; program(s) used to solve structure: *SIR2004* (Burla *et al.*, 2005 ▶); program(s) used to refine structure: *SHELXL97* (Sheldrick, 2008 ▶); molecular graphics: *DIAMOND* (Brandenburg, 2009 ▶); software used to prepare material for publication: *SHELXL97*.

## Supplementary Material

Crystal structure: contains datablock(s) I, global. DOI: 10.1107/S1600536811030832/jh2318sup1.cif

Structure factors: contains datablock(s) I. DOI: 10.1107/S1600536811030832/jh2318Isup2.hkl

Additional supplementary materials:  crystallographic information; 3D view; checkCIF report

## Figures and Tables

**Table:** 

Cu1—N2	1.9495 (16)
Cu1—O2	1.9519 (14)
Cu1—O4	1.9676 (15)
Cu1—N1	1.9682 (16)
Cu1—O1	2.3938 (15)

**Table:** 

N2—Cu1—O2	82.06 (6)
O2—Cu1—O4	89.18 (6)
N2—Cu1—N1	97.49 (7)
O4—Cu1—N1	91.15 (7)
N1—Cu1—O1	74.28 (6)

**Table 2:** Hydrogen-bond geometry (Å, °)

*D*—H⋯*A*	*D*—H	H⋯*A*	*D*⋯*A*	*D*—H⋯*A*
O4—H4*O*⋯O5	0.84	1.84	2.680 (3)	173
O4—H4*P*⋯O3^iv^	0.84	2.03	2.868 (2)	177
O5—H5*O*⋯O5^v^	0.88	2.22	2.808 (4)	124
O5—H5*P*⋯O7	0.80	1.97	2.766 (3)	171
O6—H6*O*⋯O7^vi^	0.92	1.84	2.752 (2)	177
O6—H6*P*⋯O1	0.90	1.99	2.863 (2)	163
O7—H7*O*⋯O6^vii^	0.83	1.92	2.707 (2)	157
O7—H7*P*⋯O3	0.83	2.21	3.016 (2)	166
O7—H7*P*⋯O2	0.83	2.33	2.951 (2)	132
O8—H8*O*⋯O5	0.85	1.81	2.644 (5)	167
O8—H8*P*⋯O6	0.81	2.01	2.815 (4)	174

Symmetry codes: (iv) 
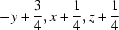
; (v) 
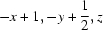
; (vi) 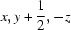; (vii) 
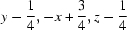
.

**Fig. 1.**
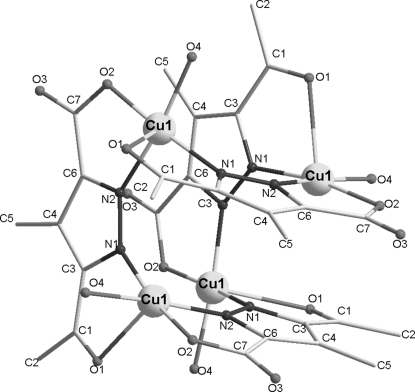
The molecular structure of the title compound. H atoms are omitted for clarity.

**Fig. 2.**
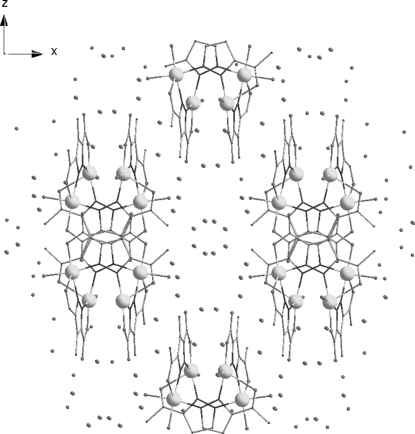
A packing diagram for the title compound, showing the columns along the y-axis direction. Copper atoms and water molecules are depicted as the big and the small balls, respectively.

## supplementary crystallographic information

### Comment

Substituted pyrazolate ligands have found widespread use as building blocks for the formation of self-assembled supramolecular coordination complexes with an array of transition metal ions and a variety of different structures, e.g., helical polymers (Piguet *et al.*, 1997; Krämer *et al.*, 2002), so-called [2 × 2] grids (Zhang *et al.*, 1996; Van der Vlugt *et al.*, 2008; Klingele *et al.*, 2007) and other polynuclear structures (Kovbasyuk *et al.*, 2004; Pons *et al.*, 2003). Introduction of different donor substituents in 3- and 5-positions of the pyrazole ring is still rare, and such ligands can be successully used for the obtaining of oligonuclear heterometallic species (Moroz *et al.*, 2010). Reported here is a new copper(II) complex with [2 × 2] grid-structure based on a novel asymmetric pyrazolate ligand having different substituents (the carboxylic and acetyl gropus) in 3- and 5-positions.

In the title compound, (I), the tetranuclear [2 × 2] grid-type complex with S4 symmetry are composed of four Cu^II^ ions, four ligands and four metal-bound water molecules (Fig. 1).

Each copper ion is nested in a square-pyramidal environment that is composed of the pyrazolate-N2, deprotonated carboxyl-O2 from a compartment of one ligand molecule and acetyl-O1 atoms, the pyrazolate-N1 from another ligand and one water-O4.

The intermetallic separations pyrazolate-bridged Cu^II^ ions is 4.0600 (4) Å which is similar to that seen in the structures reported by Zhang *et al.*, 1996 (4.098 – 4.115 Å), while the distance between diagonal copper atoms is 5.0814 (5) Å, which is more longer to that observed in the structures reported by Klingele *et al.*, 2007 (4.7091 (5) Å) and Van der Vlugt *et al.*, 2008 (4.2308 (6) Å).

The coordinated pyrazolate ligand exhibits C—C, C—N, N—N bond lengths which are normal for bridging pyrazolate rings (Sliva *et al.*, 1997; Świątek-Kozłowska *et al.*, 2000; Mokhir *et al.*, 2002). The C—O bond lengths in the deprotonated carboxylic groups differs significantly (1.239 (2) and 1.292 (2) ) which is typical for monodentately coordinated carboxylates (Wörl *et al.*, 2005*a*,*b*).

A part of the crystal packing of (I) is presented in Fig.2. In the crystal packing the complex molecules are associated via intermolecular hydrogen bonds that involve the O—H interactions between the coordinated and the solvate water molecules and the non-coordinating carboxylate-O atoms. Thus, the tetranuclear molecules are stacked along the crystallographic *x* and *y* axises, forming the columns. The columns bisect one another at right angles to give a layer-like structure.

### Experimental

The ligand 5-acetyl-4-methyl-1H-pyrazole-3-carboxylic acid (Sachse *et al.*, 2008) (0.25 g, 1.5 mmol) was added to a solution of Cu(Ac)_2_.H_2_O (0.30 g, 1.5 mmol) in H_2_O–CH_3_OH (50 mL) [80:20 v/v]. The reaction mixture was heated for 30 min with constant stirring at 80 °C until completedissolution of the ligand occurred. The resulting deep blue solution was filtered to remove any undissolved ligand and left at room temperature. Square block dark blue crystals suitable for X-ray diffraction were isolated after standing for several days (yield 0.32 g, 80%). Elemental analysis calc. (%) for C_28_H_40_Cu_4_N_8_O_20_: C 31.64; H 3.79; N 10.54; found: C 31.22; H 3.47; N 10.34.

### Refinement

The O—H and N—H hydrogen atoms were located from the difference Fourier map, and refined with *U*_iso_ = 1.5 *U*_eq_(parent atom). The remaining H atoms were positioned geometrically and were constrained to ride on their parent atoms with C—H = 0.96–0.97 Å, and with *U*_iso_ = 1.2–1.5 *U*_eq_(parent atom).

### Crystal data

**Table:**

[Cu_4_(C_7_H_6_N_2_O_3_)_4_(H_2_O)_4_]·14H_2_O	*D*_x_ = 1.638 Mg m^−^^3^
*M**_r_* = 1243.00	Mo *K*α radiation, λ = 0.71073 Å
Tetragonal, *I*4_1_/*a*	Cell parameters from 9905 reflections
Hall symbol: -I 4ad	θ = 2.6–30.3°
*a* = 13.8502 (7) Å	µ = 1.76 mm^−^^1^
*c* = 26.280 (3) Å	*T* = 100 K
*V* = 5041.1 (6) Å^3^	Block, blue
*Z* = 4	0.35 × 0.25 × 0.15 mm
*F*(000) = 2560	

### Data collection

**Table:**

Bruker SMART APEXII CCD diffractometer	3993 independent reflections
Radiation source: fine-focus sealed tube	3254 reflections with *I* > 2σ(*I*)
flat graphite crystal	*R*_int_ = 0.037
Detector resolution: 16 pixels mm^-1^	θ_max_ = 30.9°, θ_min_ = 1.7°
φ scans and ω scans	*h* = −19→19
Absorption correction: multi-scan (*SADABS*; Sheldrick, 2008)	*k* = −19→19
*T*_min_ = 0.578, *T*_max_ = 0.778	*l* = −37→38
37816 measured reflections	

### Refinement

**Table:**

Refinement on *F*^2^	Primary atom site location: structure-invariant direct methods
Least-squares matrix: full	Secondary atom site location: difference Fourier map
*R*[*F*^2^ > 2σ(*F*^2^)] = 0.034	Hydrogen site location: inferred from neighbouring sites
*wR*(*F*^2^) = 0.097	H-atom parameters constrained
*S* = 1.06	*w* = 1/[σ^2^(*F*_o_^2^) + (0.0463*P*)^2^ + 11.3899*P*] where *P* = (*F*_o_^2^ + 2*F*_c_^2^)/3
3993 reflections	(Δ/σ)_max_ < 0.001
165 parameters	Δρ_max_ = 1.20 e Å^−^^3^
0 restraints	Δρ_min_ = −0.58 e Å^−^^3^

### Special details

**Table:**

Geometry. All esds (except the esd in the dihedral angle between two l.s. planes) are estimated using the full covariance matrix. The cell esds are taken into account individually in the estimation of esds in distances, angles and torsion angles; correlations between esds in cell parameters are only used when they are defined by crystal symmetry. An approximate (isotropic) treatment of cell esds is used for estimating esds involving l.s. planes.
Refinement. Refinement of F^2^ against ALL reflections. The weighted R-factor wR and goodness of fit S are based on F^2^, conventional R-factors R are based on F, with F set to zero for negative F^2^. The threshold expression of F^2^ > 2sigma(F^2^) is used only for calculating R-factors(gt) etc. and is not relevant to the choice of reflections for refinement. R-factors based on F^2^ are statistically about twice as large as those based on F, and R- factors based on ALL data will be even larger.

### Fractional atomic coordinates and isotropic or equivalent isotropic displacement parameters (Å^2^)

**Table:**

	*x*	*y*	*z*	*U*_iso_*/*U*_eq_	Occ. (<1)
Cu1	0.164669 (16)	0.330841 (17)	0.089037 (8)	0.01628 (8)	
O1	0.19490 (11)	0.47600 (11)	0.13584 (6)	0.0220 (3)	
O2	0.18857 (10)	0.33808 (11)	0.01594 (5)	0.0191 (3)	
O3	0.11824 (11)	0.36118 (11)	−0.05924 (5)	0.0207 (3)	
O4	0.30114 (11)	0.29660 (13)	0.09939 (6)	0.0290 (4)	
H4O	0.3390	0.2983	0.0745	0.044*	
H4P	0.3283	0.3161	0.1259	0.044*	
O5	0.42451 (14)	0.31765 (18)	0.02160 (7)	0.0496 (6)	
H5O	0.4877	0.3162	0.0229	0.074*	
H5P	0.3994	0.3042	−0.0049	0.074*	
O6	0.34700 (12)	0.60006 (12)	0.10161 (6)	0.0300 (3)	
H6O	0.3363	0.6619	0.0904	0.045*	
H6P	0.2916	0.5694	0.1089	0.045*	
O7	0.32202 (12)	0.28601 (12)	−0.06718 (6)	0.0275 (3)	
H7O	0.3430	0.3107	−0.0939	0.041*	
H7P	0.2651	0.2997	−0.0606	0.041*	
O8	0.4789 (2)	0.4970 (4)	0.04080 (17)	0.0610 (16)	0.50
H8O	0.4561	0.4429	0.0310	0.092*	0.50
H8P	0.4390	0.5278	0.0564	0.092*	0.50
N1	0.13450 (12)	0.30012 (11)	0.16041 (6)	0.0161 (3)	
N2	0.03441 (11)	0.36571 (11)	0.06776 (6)	0.0152 (3)	
C1	0.17729 (14)	0.46349 (14)	0.18133 (8)	0.0191 (4)	
C2	0.18659 (19)	0.54292 (15)	0.21907 (9)	0.0284 (5)	
H2A	0.2408	0.5293	0.2419	0.043*	
H2B	0.1269	0.5478	0.2389	0.043*	
H2C	0.1982	0.6040	0.2012	0.043*	
C3	0.14765 (13)	0.36668 (13)	0.19793 (7)	0.0159 (3)	
C4	−0.07281 (13)	0.38673 (13)	0.00422 (7)	0.0161 (3)	
C5	−0.11564 (15)	0.39273 (16)	−0.04787 (7)	0.0218 (4)	
H5A	−0.0653	0.3807	−0.0733	0.033*	
H5B	−0.1430	0.4572	−0.0531	0.033*	
H5C	−0.1667	0.3442	−0.0513	0.033*	
C6	0.02326 (13)	0.36773 (13)	0.01655 (7)	0.0145 (3)	
C7	0.11406 (13)	0.35477 (13)	−0.01228 (7)	0.0159 (3)	

### Atomic displacement parameters (Å^2^)

**Table:**

	*U*^11^	*U*^22^	*U*^33^	*U*^12^	*U*^13^	*U*^23^
Cu1	0.01545 (12)	0.02178 (13)	0.01160 (11)	−0.00004 (8)	−0.00341 (8)	0.00135 (8)
O1	0.0244 (7)	0.0211 (7)	0.0207 (7)	−0.0033 (5)	−0.0084 (5)	0.0037 (5)
O2	0.0159 (6)	0.0270 (7)	0.0145 (6)	−0.0023 (5)	−0.0004 (5)	0.0028 (5)
O3	0.0246 (7)	0.0253 (7)	0.0122 (6)	−0.0018 (6)	0.0017 (5)	0.0031 (5)
O4	0.0222 (7)	0.0459 (10)	0.0190 (7)	0.0079 (7)	−0.0080 (6)	−0.0070 (7)
O5	0.0298 (9)	0.0881 (17)	0.0307 (9)	0.0203 (10)	−0.0046 (8)	−0.0200 (10)
O6	0.0302 (8)	0.0300 (8)	0.0297 (8)	−0.0047 (7)	−0.0066 (7)	0.0033 (7)
O7	0.0244 (7)	0.0294 (8)	0.0288 (8)	−0.0005 (6)	0.0089 (6)	0.0036 (6)
O8	0.0102 (14)	0.102 (4)	0.071 (3)	0.0179 (18)	−0.0138 (16)	−0.068 (3)
N1	0.0212 (8)	0.0149 (7)	0.0121 (7)	0.0011 (6)	−0.0041 (6)	−0.0001 (5)
N2	0.0158 (7)	0.0197 (7)	0.0102 (6)	−0.0028 (5)	−0.0003 (5)	0.0028 (5)
C1	0.0199 (8)	0.0157 (8)	0.0216 (9)	0.0016 (6)	−0.0083 (7)	−0.0002 (7)
C2	0.0416 (13)	0.0164 (9)	0.0270 (10)	−0.0008 (8)	−0.0070 (9)	−0.0035 (8)
C3	0.0188 (8)	0.0150 (8)	0.0138 (8)	0.0031 (6)	−0.0046 (6)	−0.0008 (6)
C4	0.0172 (8)	0.0170 (8)	0.0140 (8)	−0.0046 (6)	−0.0030 (6)	0.0045 (6)
C5	0.0218 (9)	0.0284 (10)	0.0152 (8)	−0.0034 (8)	−0.0070 (7)	0.0038 (7)
C6	0.0164 (8)	0.0158 (8)	0.0112 (7)	−0.0037 (6)	−0.0017 (6)	0.0029 (6)
C7	0.0184 (8)	0.0155 (8)	0.0138 (8)	−0.0037 (6)	0.0009 (6)	0.0025 (6)

### Geometric parameters (Å, °)

**Table:**

Cu1—N2	1.9495 (16)	O8—H8O	0.8529
Cu1—O2	1.9519 (14)	O8—H8P	0.8080
Cu1—O4	1.9676 (15)	N1—N2^i^	1.329 (2)
Cu1—N1	1.9682 (16)	N1—C3	1.362 (2)
Cu1—O1	2.3938 (15)	N2—N1^ii^	1.329 (2)
Cu1—Cu1^i^	4.0600 (4)	N2—C6	1.355 (2)
Cu1—Cu1^ii^	4.0600 (4)	C1—C3	1.469 (3)
Cu1—Cu1^iii^	5.0814 (5)	C1—C2	1.487 (3)
O1—C1	1.232 (3)	C2—H2A	0.9800
O2—C7	1.292 (2)	C2—H2B	0.9800
O3—C7	1.239 (2)	C2—H2C	0.9800
O4—H4O	0.8400	C3—C4^i^	1.405 (3)
O4—H4P	0.8355	C4—C6	1.394 (2)
O5—H5O	0.8760	C4—C3^ii^	1.405 (3)
O5—H5P	0.7998	C4—C5	1.494 (3)
O6—H6O	0.9174	C5—H5A	0.9800
O6—H6P	0.8985	C5—H5B	0.9800
O7—H7O	0.8324	C5—H5C	0.9800
O7—H7P	0.8289	C6—C7	1.479 (3)
			
N2—Cu1—O2	82.06 (6)	H7O—O7—H7P	114.5
N2—Cu1—O4	171.24 (6)	H8O—O8—H8P	111.3
O2—Cu1—O4	89.18 (6)	N2^i^—N1—C3	108.05 (15)
N2—Cu1—N1	97.49 (7)	N2^i^—N1—Cu1	130.03 (12)
O2—Cu1—N1	170.07 (6)	C3—N1—Cu1	121.00 (13)
O4—Cu1—N1	91.15 (7)	N1^ii^—N2—C6	108.91 (15)
N2—Cu1—O1	95.81 (6)	N1^ii^—N2—Cu1	137.70 (12)
O2—Cu1—O1	115.65 (6)	C6—N2—Cu1	113.30 (12)
O4—Cu1—O1	87.90 (6)	O1—C1—C3	118.16 (17)
N1—Cu1—O1	74.28 (6)	O1—C1—C2	121.73 (18)
N2—Cu1—Cu1^i^	94.38 (5)	C3—C1—C2	120.11 (18)
O2—Cu1—Cu1^i^	123.35 (4)	C1—C2—H2A	109.5
O4—Cu1—Cu1^i^	90.48 (5)	C1—C2—H2B	109.5
N1—Cu1—Cu1^i^	46.73 (5)	H2A—C2—H2B	109.5
O1—Cu1—Cu1^i^	120.95 (4)	C1—C2—H2C	109.5
N2—Cu1—Cu1^ii^	44.57 (5)	H2A—C2—H2C	109.5
O2—Cu1—Cu1^ii^	125.84 (4)	H2B—C2—H2C	109.5
O4—Cu1—Cu1^ii^	144.00 (5)	N1—C3—C4^i^	109.93 (16)
N1—Cu1—Cu1^ii^	55.97 (5)	N1—C3—C1	116.13 (16)
O1—Cu1—Cu1^ii^	70.56 (4)	C4^i^—C3—C1	133.70 (17)
Cu1^i^—Cu1—Cu1^ii^	77.482 (5)	C6—C4—C3^ii^	103.01 (15)
N2—Cu1—Cu1^iii^	43.82 (5)	C6—C4—C5	127.02 (18)
O2—Cu1—Cu1^iii^	100.07 (4)	C3^ii^—C4—C5	129.97 (17)
O4—Cu1—Cu1^iii^	139.12 (6)	C4—C5—H5A	109.5
N1—Cu1—Cu1^iii^	73.39 (5)	C4—C5—H5B	109.5
O1—Cu1—Cu1^iii^	121.81 (4)	H5A—C5—H5B	109.5
Cu1^i^—Cu1—Cu1^iii^	51.259 (3)	C4—C5—H5C	109.5
Cu1^ii^—Cu1—Cu1^iii^	51.259 (3)	H5A—C5—H5C	109.5
C1—O1—Cu1	110.22 (12)	H5B—C5—H5C	109.5
C7—O2—Cu1	116.03 (12)	N2—C6—C4	110.08 (16)
Cu1—O4—H4O	119.0	N2—C6—C7	114.16 (15)
Cu1—O4—H4P	118.0	C4—C6—C7	135.66 (17)
H4O—O4—H4P	111.1	O3—C7—O2	123.20 (17)
H5O—O5—H5P	117.6	O3—C7—C6	122.78 (17)
H6O—O6—H6P	111.8	O2—C7—C6	114.02 (15)

Symmetry codes: (i) *y*−1/4, −*x*+1/4, −*z*+1/4; (ii) −*y*+1/4, *x*+1/4, −*z*+1/4; (iii) −*x*, −*y*+1/2, *z*.

### Hydrogen-bond geometry (Å, °)

**Table:**

*D*—H···*A*	*D*—H	H···*A*	*D*···*A*	*D*—H···*A*
O4—H4O···O5	0.84	1.84	2.680 (3)	173.
O4—H4P···O3^iv^	0.84	2.03	2.868 (2)	177.
O5—H5O···O5^v^	0.88	2.22	2.808 (4)	124.
O5—H5P···O7	0.80	1.97	2.766 (3)	171.
O6—H6O···O7^vi^	0.92	1.84	2.752 (2)	177.
O6—H6P···O1	0.90	1.99	2.863 (2)	163.
O7—H7O···O6^vii^	0.83	1.92	2.707 (2)	157.
O7—H7P···O3	0.83	2.21	3.016 (2)	166.
O7—H7P···O2	0.83	2.33	2.951 (2)	132.
O8—H8O···O5	0.85	1.81	2.644 (5)	167.
O8—H8P···O6	0.81	2.01	2.815 (4)	174.

Symmetry codes: (iv) −*y*+3/4, *x*+1/4, *z*+1/4; (v) −*x*+1, −*y*+1/2, *z*; (vi) *x*, *y*+1/2, −*z*; (vii) *y*−1/4, −*x*+3/4, *z*−1/4.
